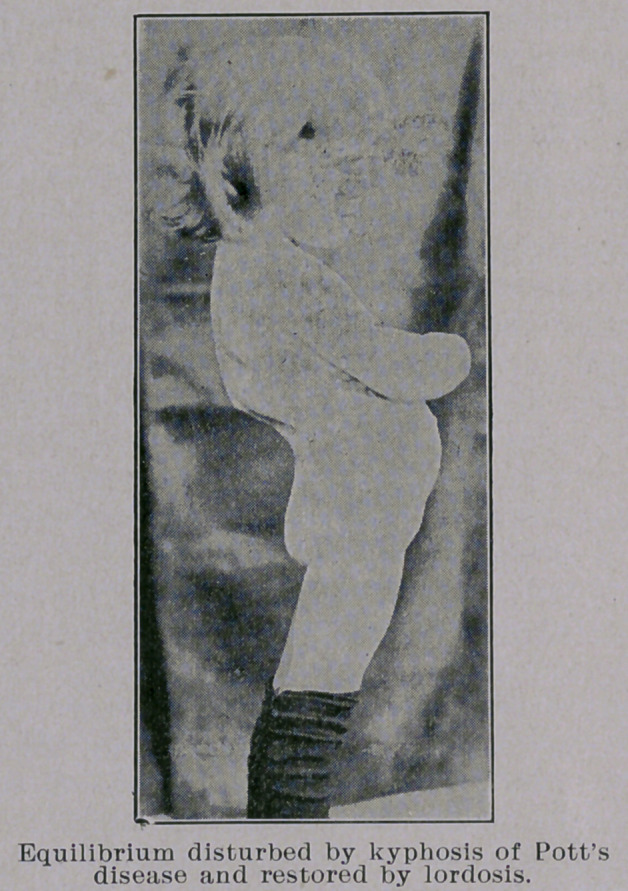# New York Academy of Medicine

**Published:** 1900-12

**Authors:** 


### New York Academy of Medicine.

### SECTION ON ORTHOPEDIC SURGERY.

(Meeting of October 19, 1900.)

Dr. H. Gibney read a paper on the "Diagnosis of Pott's Disease." The paper was illustrated by the exhibition of photographs and the presentation of patients.

CASE I. *Cervical Pott's Disease.*—Girl 8 years of age. Marked deformity from disease of long duration of several of the cervical vertebræ with scars of abscesses below the site of the disease. Treatment had been discontinued in the summer of 1900. The child had worn a head support combined at first with a plaster of Paris jacket, and afterwards with a Knight's spinal brace.

Pain near the seat of the disease, which is often absent in the other regions, is a common symptom in this region, with a sensitive area at the side of the neck, severe pain with voluntary motion of the head and neck and apparent torticollis yielding easily to traction applied in such a manner as to hold the head in its normal position. Before treatment, relief was sought by a supporting hand held under the chin. Abscesses are not an uncommon incident of cervical disease, detected by an examination of the posterior wall of the pharynx or burrowing under the superficial muscles of the neck.

CASE II. *Cervical and Dorsal.*—Boy 5 years of age. Affected for two and one-half years with disease extending from the middle cervical to the middle dorsal region. Two abscesses had opened spontaneously at the sides of the neck under the sterno-cleido-mastoid muscle. He had worn a plaster of Paris jacket and a jury-mast for eighteen months.

A grunting noise with each expiration is almost characteristic of caries of the dorsal region, and an early diagnosis is greatly assisted by the occurrence of gastralgia and the appearance of a careful gait and a peculiar apprehensive attitude, expressive of timidity and insecurity, and an instinctive desire to avoid disturbance of the diseased vertebræ. The first sign of a kyphos is seen in a slight angle breaking the long natural curve of the spinous processes observed in profile as the patient lies prone.

CASE III. *Tenth Dorsal—First Stage.*—Girl 8 years of age. Under observation since May 5, 1900, and regarded for a time as a case of lateral curvature with a hyper-sensitive, almost neuralgic, condition of the spine. Very recently a suspicious point had been detected at the tenth dorsal and treatment would now be by a Knight's support.

Dr. T. H. Myers said that lateral curvature often attended incipient Pott's disease and obscured the nature of the more serious affection, as had occurred in the present instance. He thought that these doubtful cases should be considered as caries of the vertebræ until a positive diagnosis could be made.

Dr. H. S. Stokes said that in obscure cases of early Pott's disease the plaster of Paris jacket was valuable as a means of verifying the diagnosis. In cases in which there was at first no apparent deformity if the jacket were applied and left on for a time, then removed, the kyphosis, if present, would be seen at once. This effect was seen too soon to be due to further progress of the disease, nor could it be said that the jacket had caused the kyphosis. In a doubtful case, showing no deformity, he would apply the jacket as a diagnostic measure.

Dr. A. B. Judson said that similarly the tumor of white swelling of the knee became more obvious soon after the beginning of mechanical treatment probably from pressure and restraint applied to the soft parts.

Dr. Gibney resumed his presentation of patients as follows:

CASE IV. *Dorso-Lumbar.*—Girl 2½ years old. Affected with disease of the dorso-lumbar region of nine months' duration. No abscesses. The spine had the marked rigidity which attended disease in this region and marked gastralgia had been a part of the history of the case. A plaster of Paris jacket had been applied at first, but lately a recession of the deformity had been observed to follow the strict application of a Bradford frame.

CASE V. *Eleventh Dorsal—Third Lumbar.*—Girl 13 years old, who had recently come from Russia with a very marked kyphos. But little had been learned of the history and treatment. Sinuses were discharging at points where abscesses had opened spontaneously. The gait and attitude were very characteristic of disease in this region. A Knight's support had been applied, and as the child's general condition was fair, the prognosis was good.

Dr. Myers said that the characteristic attitudes of Pott's disease, although early and important signs, were also seen in osteitis of a syphilitic or malignant origin. It was, therefore, important to consider the personal and family history, the age, the location of the disease and the mode of onset as well as the pain and tenderness. The fourth patient presented had been free from pain in the abdomen and legs. Pain in the terminations of the nerves was not so early or so prominent a symptom in the lumbar as in the dorsal region, while local tenderness was more apt to be recognized in the cervical region, where the affected parts could be more easily palpated than in the other spinal regions. In the cervical region the vertebral articulations might become infected by organisms gaining access from the pharynx after measles or scarlet fever with resulting muscular spasms and malpositions of the head simulating those of Pott's disease, and it might be a long time before it could be decided that a post-pharyngeal abscess had its origin in vertebral caries. A long time might also elapse before it could be known that a traumatic osteitis in the cervical or lumbar region had become tubercular. There were absolutely no pathognomonic symptoms.

Dr. J. P. Fiske said that he had not as yet seen a case of traumatic spine go on to tubercular caries.

Dr. Judson said that Pott's disease presented some unexpected features, such as the occurrence of pain in the front of the trunk while the disease was in the back. Some patients also with serious and purulent destruction of bone maintained the appearance and general ability of robust health. This affection, justly compared with fracture of a central and most important part of the skeleton, was, as a rule, so free from local pain and disability that when these symptoms were persistent and exaggerated Pott's disease gave way to malignant disease of the vertebræ as a probable diagnosis.

Dr. Myers said that the diagnosis of the latter affection would be assisted by consulting the following clinical features: Rapid emaciation and loss of strength, every motion exquisitely painful, pain constant but motor paralysis less constant, marked muscular rigidity, kyphosis absent or late in its appearance, occurrence at any age.

Dr. Fiske said that as they all had deformity the presentation of these patients failed to throw light on the most important question, that of making an early diagnosis. Diagnosis before deformity was an extremely difficult thing, and proportionately important and desirable. Suspicious spinal symptoms might be produced by rheumatism, by neurotic reflexes, myosites following a blow or by some other and more obscure muscular lesion. He had seen cases in which circumcision had dissipated spinal symptoms which had been hard to interpret. Muscular spasm or spinal rigidity could not alone support a diagnosis of tuberculosis of the spine.

Dr. C. R. L. Putnam recalled the history of a case which he had observed in a foreign hospital. A man, 45 years of age, totally paraplegic, was thought to have disease of the first and second lumbar vertebræ with a tubercular abscess pressing on the spinal cord. The removal of two laminæ revealed the presence of an echinicoccus cyst behind the theca. The result was unfavorable.

Dr. Myers had seen a tumor of the lower cervical cord produce not only symptoms of pressure on the cord, but also the local pain and muscular rigidity which usually attend vertebral disease.

Dr. F. A. Goodwin, of Susquehanna, Pennsylvania, said that railway brakemen, from their custom of jumping off and on trains in motion, frequently received spinal injuries accompanied by rigidity, pain on pressure and other symptoms of true Pott's disease. Perfect rest for a long time, however, almost always cleared up the diagnosis. It had been his misfortune to see a number of patients in whom the diagnosis of Pott's disease had been inexcusably postponed by eminent authorities. He instanced the history of a little boy who had been treated for asthma and other affections without an examination for kyphosis which had existed to a marked degree for a long time, during which grunting expiration, pain, inability to stoop and rigidity of the spine had been obvious features of the case. On the other hand, he had made a diagnosis of Pott's disease in a little girl who had a board-like rigidity of the spine. She could not stoop to pick up a coin from the floor without putting a hand on the knee for support. Her recovery without treatment was explicable by the supposition that there had been synovitis of the costo-vertebral and costo-transverse articulations. He thought that a diagnosis before the appearance of deformity was exceptional, and recognized the inherent difficulties of the situation.

Dr. L. W. Ely referred to the opinion which prevailed among general practitioners that Pott's disease in the dorsal and lumbar regions was attended by sensitiveness to pressure on the spinous processes. Although this supposition was not unreasonable, in view of the nature of the lesion, the fact was that this symptom was of very rare occurrence. Running the fingers down the spinous processes in a doubtful case was of almost no value in making a diagnosis.

Dr. G. R. Elliott said that in a rapid carious process we had the full quota of symptoms clearly defined while a slow morbid action gave but few and obscure indications. The X ray had been a disappointment in this field. It had failed to reveal a deposit before the appearance of deformity. What was desired was an early diagnosis, a diagnosis before deformity which, of itself, made the diagnosis without the assistance of symptoms or any other signs. A most important early symptom was abdominal pain. How often are we told of the postponement of a spinal examination in favor of treatment for intestinal disturbance until an early diagnosis was impossible. A child should be examined with all the clothing removed. In no other way could the obscure signs be recognized. The enlarged abdomen was another important early sign. The contraction of a psoas muscle, exposing one to the risk of a faulty diagnosis of hip disease, might be the earliest sign of Pott's disease. He recalled the case of a child who was said to have cervical caries of two months' duration following scarlet fever with rheumatism. There was painful spasm of the muscles of the neck, the head resting on the shoulder and a hand supporting the chin. The symptoms all disappeared without fixation after treatment by simple suspension. On the other hand, a patient with supposed rheumatism of the spine, whose symptoms included pain in the back, stiffness and misunderstood reflex spasm, was bathed, rubbed and shaken up for three months, and after vigorous anti-rheumatic treatment had lasted for a year, the appearance of kyphosis determined the diagnosis.

Dr. Gibney said that photographs clearly presented the attitudes but failed to display the characteristic movements and deportment of the patient affected with Pott's disease. There was in his collection, however, one which graphically copied (see accompanying figure) the over-erect attitude which was assumed by the patient's

Equilibrium disturbed by kyphosis of Pott's disease and restored by lordosis.

entire figure and threw light on the mechanism of the production of the lordosis which appeared as a compensating curve below the kyphos.

## Figures and Tables

**Figure f1:**